# Synthetic Peptide and Protein Domain Arrays Prepared by the SPOT Technology

**DOI:** 10.1002/cfg.99

**Published:** 2001-10

**Authors:** Jens Schneider-Mergener

**Affiliations:** 1 Institut für Medizinische Immunologie Universitätsklinikum Charité Humboldt Universität zu Berlin Berlin 10098 Germany; 2 Jerini AG Rudower Chausse 29 Berlin 12489 Germany

## Abstract

The SPOT^™^ technology for highly parallel synthesis of peptides on flat surfaces in array type format has evolved into a versatile toolbox for a variety of applications in proteomics such as mapping protein-protein interactions and profiling the substrate specificity of enzymes such as kinases and proteases. Originally developed for the synthesis of short overlapping peptide sequences for mapping antibody epitopes this technology has recently been extended to the synthesis of functional protein domains. This opens up a variety of future applications such as target identification and protein expression profiling.


**Comparative and Functional Genomics**
*Comp Funct Genom* 2001; **2**: 307–309.
**DOI:** 10.1002/cfg.99

**Conference Review**

### Synthetic peptide and protein domain arrays prepared by the SPOT technology

### A presentation for the ESF workshop 'Proteomics: Focus on protein interactions'

Jens Schneider-Mergener[1,2]*

[1] *Institut für Medizinische Immunologie, Universitätsklinikum Charité, Humboldt Universität zu Berlin, 10098 Berlin, Germany*
[2] *Jerini AG, Rudower Chausse 29, 12489 Berlin, Germany*

**Correspondence to:*
*J. Schneider-Mergener, Jerini AG, Rudower Chausse 29, 12489 Berlin, Germany.*
*E-mail: Schneider-mergener@jerini.de*

Received: 22 June 2001
Accepted: 27 July 2001

### Abstract

**The SPOT™ technology for highly parallel synthesis of peptides on flat surfaces in array type format has evolved into a versatile toolbox for a variety of applications in proteomics such as mapping protein-protein interactions and profiling the substrate specificity of enzymes such as kinases and proteases. Originally developed for the synthesis of short overlapping peptide sequences for mapping antibody epitopes this technology has recently been extended to the synthesis of functional protein domains. This opens up a variety of future applications such as target identification and protein expression profiling. Copyright © 2001 John Wiley & Sons, Ltd.**

**Keywords:** **peptide/protein chips; SPOT synthesis; kinase; protease; antibody; receptor**

### Introduction

In contrast to the development of DNA array technologies, the preparation and application of peptide, protein domain and protein arrays is in its very infancy. In the last few years it has become obvious that the information obtained from DNA chips is not sufficient and has to be complemented by information from the proteome. It is beyond any doubt that protein chip technologies will play a crucial role in deciphering the human proteome and identifying new target proteins for therapeutic use. Peptide/protein chips will furthermore play an essential role in the future for individualised diagnosis of the human health status, which is a prerequisite for the development and application of personalised medicines. The synthesis of peptide and protein domain arrays by the SPOT technology [4,7] is reported here, addressing the preparation and the application of these arrays in various aspects of proteomics research.

The SPOT technology involves the spatially addressed synthesis of peptide arrays on flat surfaces. It was originally developed to synthesise peptide scans on cellulose membranes for the purpose of antibody epitope mapping [4,8]. Since then, the SPOT technology has synthetically evolved into automated peptide and protein domain synthesis on various types of flat surfaces (cellulose, polypropylene, glass) using a variety of chemistries for attaching the compounds to the surface. This enables successive, highly parallel solid or solution phase screenings of thousands of different peptides and protein domains for biological activity, such as binding or enzymatic activity (for reviews see: [5,7,12]).

### Preparation of arrays

The SPOT technology started with synthesis on cellulose membranes, but other types of flat surfaces such as polypropylene [12] or glass (Schutkowski, unpublished; [11]) have been used recently. The compounds can be coupled by different chemistries through several types of linkers to the flat surfaces, depending on the successive application of the arrays. Very stable attachments via an ether bond, for instance, ensure the reusability of the cellulose membranes up to ten times for protein binding studies. Attachment via an ester bond or a photocleavable linker allows the selective cleavage of the

Copyright © 2001 John Wiley & Sons, Ltd.

compounds from the surface for the purpose of quality control, or application of the compounds in standard micro-titer plate assay systems [12]. Currently, densities up to 17 peptides/cm$^2$ can be prepared on a $20 \times 30$ cm cellulose membrane by stepwise SPOT synthesis in a semi- or fully automated fashion (Zerweck, unpublished). By applying printing technologies for the coupling of peptides or protein domains synthesised by conventional methods, much higher densities ($>1000$) can be achieved, especially on non-porous glass materials [6]; (Reimer and Schutkowski, unpublished).

### Types of molecules

A variety of peptide molecules can be prepared by the SPOT technique (Figure 1). Linear peptides can routinely be synthesised up to a length of 20 amino acids, the quality of which is similar compared to standard methods [12]. In addition, much longer peptides have been prepared by stepwise synthesis, as shown for the synthesis of a WW protein domain consisting of 42 amino acids [9]. The synthesis is not restricted to linear L-peptides. D-enantiomers, cyclic variants and phoshorylated or other modified peptides can be readily prepared. An upcoming application will be the combination of SPOT synthesis and chemo-selective ligation techniques (for review see: [2]) for the preparation of protein domains by linking two or more peptides onto each spot. This has been demonstrated for the synthesis of several thousand variants of a WW protein domain [10]. Within the near future this should lead to molecules with lengths of up to 100 amino acids.

### Applications of arrays

Numerous applications of synthetic peptide and protein domain arrays have been reported or can be preconceived (for reviews see: [7,5,11]): 1 Mapping of protein-protein contact sites (antibody-antigen, receptor-ligand, specificities of signaling protein modules, etc.), 2 profiling of enzymatic activities (kinases, proteases, transferases, etc.), 3 protein expression profiling or 4 deciphering signal transduction pathways. For the future applications, 3 and 4, the chips have to be incubated with cell lysates [1] or even whole cells [3]. Combining the SPOT technology with modern techniques in mass spectrometry will allow a highly parallel identification of known or unknown proteins that bind to distinct un/modified peptides or protein domains. Another major application of peptide and protein

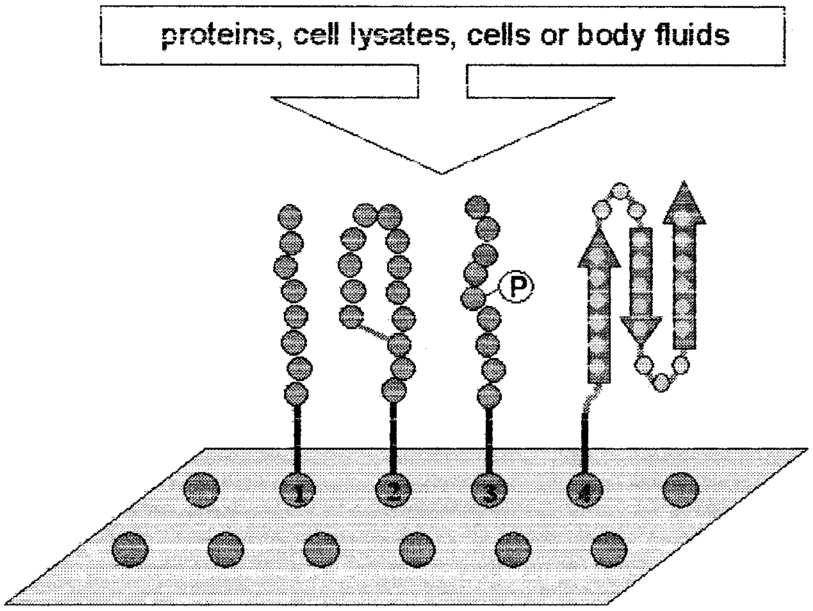

**Figure .** Different types of peptides can be synthesised either by stepwise manner or printed as arrays: (1) short linear peptides ($<20$ mers), (2) peptides cyclised by different chemical strategies, (3) modified peptides (phosphorylated, glycosylated etc.) and (4) protein domains (WW, SH2, SH3, PDZ etc.)

Copyright © 2001 John Wiley & Sons, Ltd.

chips will be the parallel diagnosis of human diseases leading to detailed pictures of the health status of single individuals.

### References

1. Adam-Klages S, Adam D, Wiegmann K, *et al.* 1996. The novel human TNF-receptor p55-associated WD-repeat protein FAN mediates neutral SMase activation. *Cell* **86**: 937–947.
2. Dawson PE, Kent SBH. 2000. Synthesis of native proteins by chemical ligation. *Ann Rev Biochem* **69**: 923–960.
3. Falsey JR, Renil M, Park S, Li S, Lam KS. 2001. Peptide and small molecule microarray for high throughput cell adhesion and functional assays. *Bioconjug Chem* **12**: 346–353.
4. Frank R. 1992. Spot synthesis: an easy technique for the positionally addressable, parallel chemical synthesis on a membrane support. *Tetrahedron* **48**: 9217–9232.
5. Frank R, Schneider-Mergener J. 2001. SPOT-synthesis: scope and applications. Introduction to: *Peptide arrays on membrane supports–synthesis and applications.* Springer lab manual (Koch/Mahler eds.), in press.
6. MacBeath G, Schreiber SL. 2000. Printing proteins as microarrays for high-throughput function determination. *Science* **289**: 1760–1763.
7. Reineke U, Volkmer-Engert R, Schneider-Mergener J. 2001. Applications of peptide arrays prepared by the SPOT technology. *Curr Opin Biotech* **12**: 59–64.
8. Reineke U, Kramer A, Schneider-Mergener J. 1999. Knowledge- and library-based mapping of discontinuous protein-protein-interactions by spot synthesis. *Curr Top Microbiol Immunol.* **243**: 23–36.
9. Töpert F, Pires R, Landgraf C, Oschkinat H, Schneider-Mergener J. 2001a. Synthesis of an array comprising 837 variants of the hYAP WW protein domain. *Angew Chem Int Ed Engl* **40**: 897–900.
10. Töpert F, Knaute T, Guffler S, Schneider-Mergener J. 2001b. Incorporating new ligand specificities into a 38mer WW protein domain applying an array of 6859 WW domain variants prepared by a combination of SPOT synthesis and native chemical ligation. Proceedings of the 17$^{th}$ American Peptide Symposium, in press.
11. Website Jerini AG. For an extensive overview of references on applications of the SPOT technology and recent developments in peptide microarrays: **http://www.jerini.com**
12. Wenschuh H, Volkmer-Engert R, Schmidt M, Schulz M, Schneider-Mergener J, Reineke U. 2000. Coherent membrane supports for parallel micro-synthesis of bioactive peptides. *Biopolymers* **55**: 188–206.

Copyright © 2001 John Wiley & Sons, Ltd.
